# The Brillouin gain of vector modes in a few-mode fiber

**DOI:** 10.1038/s41598-017-01621-7

**Published:** 2017-05-08

**Authors:** Prabin Pradhan, Dipankar Sengupta, Lixian Wang, Christine Tremblay, Sophie LaRochelle, Bora Ung

**Affiliations:** 1 École de technologie supérieure, Department of Electrical Engineering, Montreal, H3C 1K3 Canada; 2 Center for Optics, Photonics and Lasers (COPL), Université Laval, Québec, QC G1V 0A6 Canada

## Abstract

In this work, we demonstrate the measurement of the Brillouin gain spectra of vector modes in a few-mode fiber for the first time using a simple heterodyne detection technique. A tunable long period fiber grating is used to selectively excite the vector modes supported by the few-mode fiber. Further, we demonstrate the non-destructive measurement of the absolute effective refractive indices (*n*_*eff*_) of vector modes with ~10^−4^ accuracy based on the acquired Brillouin frequency shifts of the modes. The proposed technique represents a new tool for probing and controlling vector modes as well as modes carrying orbital angular momentum in optical fibers with potential applications in advanced optical communications and multi-parameter fiber-optic sensing.

## Introduction

Recently, space division multiplexing (SDM) has been proposed to overcome the looming capacity crunch in conventional single mode fibers (SMFs). In order to enable SDM in practice, many different types of specialty fibers have been devised^1^. In particular, the few-mode fiber (FMF) has attracted a lot of attention due to its potential application in optical telecommunication and fiber sensing applications. Few-mode fibers are particularly promising for mode-division multiplexing^2^, where multiple information channels can be transmitted across independent spatial modes with minimum crosstalk, thereby enhancing the data carrying capacity inside a single core fiber by many folds. Moreover, recent research activities have reported promising results in the development of multi-parameter and distributed sensors based on the LP modes of FMFs^3–5^. Both fields of application rely on harnessing the rich modal diversity in FMFs. In order to fully exploit this modal diversity, the nontrivial modal properties of the FMF must be characterized accurately. Specifically, the effective refractive indices (ERIs) of the modes and ERI differences (Δ*n*_*eff*_) between adjacent modes represent the key characteristics of FMFs that determine whether stable (i.e. low intermodal cross-talk) transmission of multiple discrete modes is possible in practice.

Several characterization methods and tools have been developed for single mode fibers^6, 7^ but, in the case of FMFs, new technical challenges arise due to the co-existence of multiple co-propagating modes. Some of the recent characterization techniques on FMF include the phase-shift method^8^, the S2 method^9^, the time of flight method^10^, the microwave interferometric technique^11^ and the optical low-coherence interferometry method^12^. However, most of the work done on FMFs have been limited to the measurements of the scalar LP mode groups and have so far neglected the underlying vector modes, which require delicate spectral and spatial control in order to be detected. Vector modes in optical fibers constitute the fundamental basis set of LP modes (in the scalar approximation) as well as of modes carrying orbital angular momentum (OAM) which represent another potential avenue for SDM based communications^13^. Therefore, fundamental information and control over the vector modes is critical for future SDM fiber communication links and OAM based fiber-optic sensors^14^. A recent characterization technique of vector modes based on permanently inscribed fiber Bragg gratings (FBGs) has been demonstrated^15^. While this technique is effective, the accuracy is limited by the perturbation induced by the refractive index modulation of the FBG, and its longitudinal spatial resolution and coverage are restricted by the length and number of FBGs present in the fiber under test.

Recent works performed on FMFs have highlighted the potential of exploiting the nonlinear stimulated Brillouin scattering in such fibers towards SDM^16, 17^ and fiber-optic sensing applications^3, 18^. In this study, we report the non-destructive nonlinear characterization of vector modes in FMF – excited via a tunable long period fiber grating (LPFG) – by means of their Brillouin gain spectrum (BGS). We show how the BGS of the vector modes in FMF can be independently measured and analyzed, and how the corresponding ERIs of the vector modes can be subsequently retrieved from the data.

## Experiment

The few-mode fiber used in the experiment is an inverse parabolic graded-index fiber designed to mitigate the modal crosstalk between different vector modes by ensuring large Δ*n*_*eff*_ values between adjacent guided modes^19^. This FMF with 3 *μm* core radius supports the fundamental HE_11_ (even and odd) mode, the TE_01_, HE_21_ (even and odd), TM_01_, EH_11_ (even and odd), and HE_31_ (even and odd) vector modes in the C-band. We note that, in the scalar approximation, the LP_11_ mode group is created by a pairwise combination of the TE_01_, HE_21_ (even and odd) or TM_01_ modes. At 1550 nm, the FMF also supports up to 6 OAM modes through coherent combination of the degenerate high-order hybrid modes (HE_21_, EH_11_, HE_31_). The ERIs of all the vector modes can be calculated by importing the measured refractive index profile (RIP) in a full-vector finite-element method (FEM) mode solver. The minimum Δ*n*_*eff*_ value between the vector components (TE_01_, HE_21_, TM_01_) of the LP_11_ group for this FMF is expected to be in the range of 2.9 × 10^−4^ and 3.7 × 10^−4^. This large modal separation allows the stable propagation of these vector modes within the FMF. On the other hand, the proximity of the EH_11_, HE_31_ modes to modal cut-off induces very large modal losses^19^ such that we can neglect the latter higher-order vector modes. A tunable mechanical LPFG that efficiently excites the TE_01_, HE_21_ and TM_01_ mode has been designed and fabricated^20^. The LPFG is fabricated with a nominal grating period of ${{\rm{\Lambda }}}_{\circ }$ = 200 *μm*. This grating period can further be tuned by positioning the fiber at specific angles *θ* with respect to the grating lines. The angle of the grating can thus be varied from 0° (${{\rm{\Lambda }}}_{\circ }$ = 200 *μm*) to 26°, corresponding to a maximum grating period of Λ = 222 *μm*. Strong coupling of the power from the fundamental HE_11_ towards a co-propagating higher-order mode occurs when the phase matching condition *λ* = Λ · Δ*n*_*eff*_ is satisfied for a given vector mode, where *λ* is the resonant coupling wavelength, Δ*n*_*eff*_ is the ERI difference between the fundamental and high-order vector modes, and ${\rm{\Lambda }}={{\rm{\Lambda }}}_{\circ }/\cos \,\theta$, is the designed grating period.

**Figure 1:**
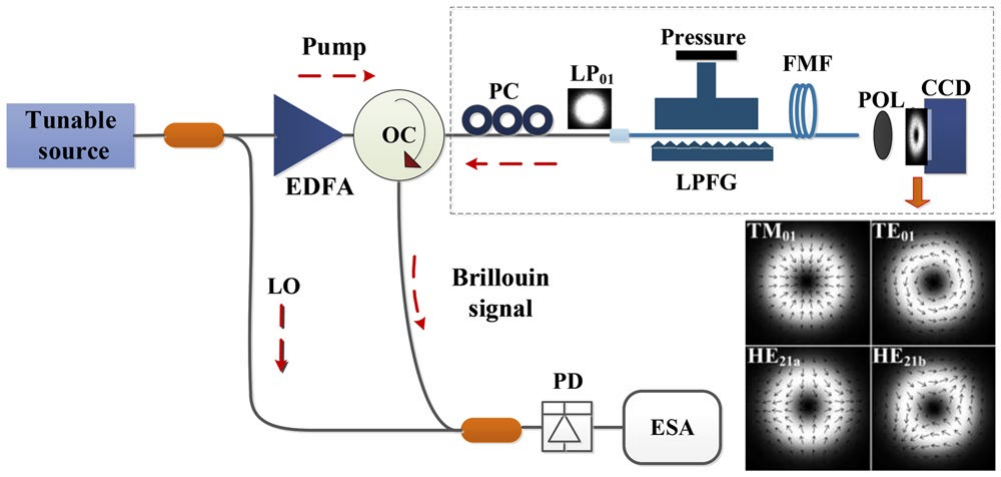
Experimental setup for the Brillouin gain spectra measurement of vector modes. (PC: polarization controller; LPFG: long period fiber grating; LO: local oscillator; FMF: few-mode fiber; POL: linear polariser; PD: photodetector; ESA: Electrical spectrum analyzer; EDFA: Erbium doped fiber amplifier; OC: optical circulator). Inset: Electric field representation (arrows) of the TM_01_, TE_01_, HE_21*a*_ and HE_21*b*_ (LP_11_ mode group) calculated using FEM^19^.

**Figure 2:**
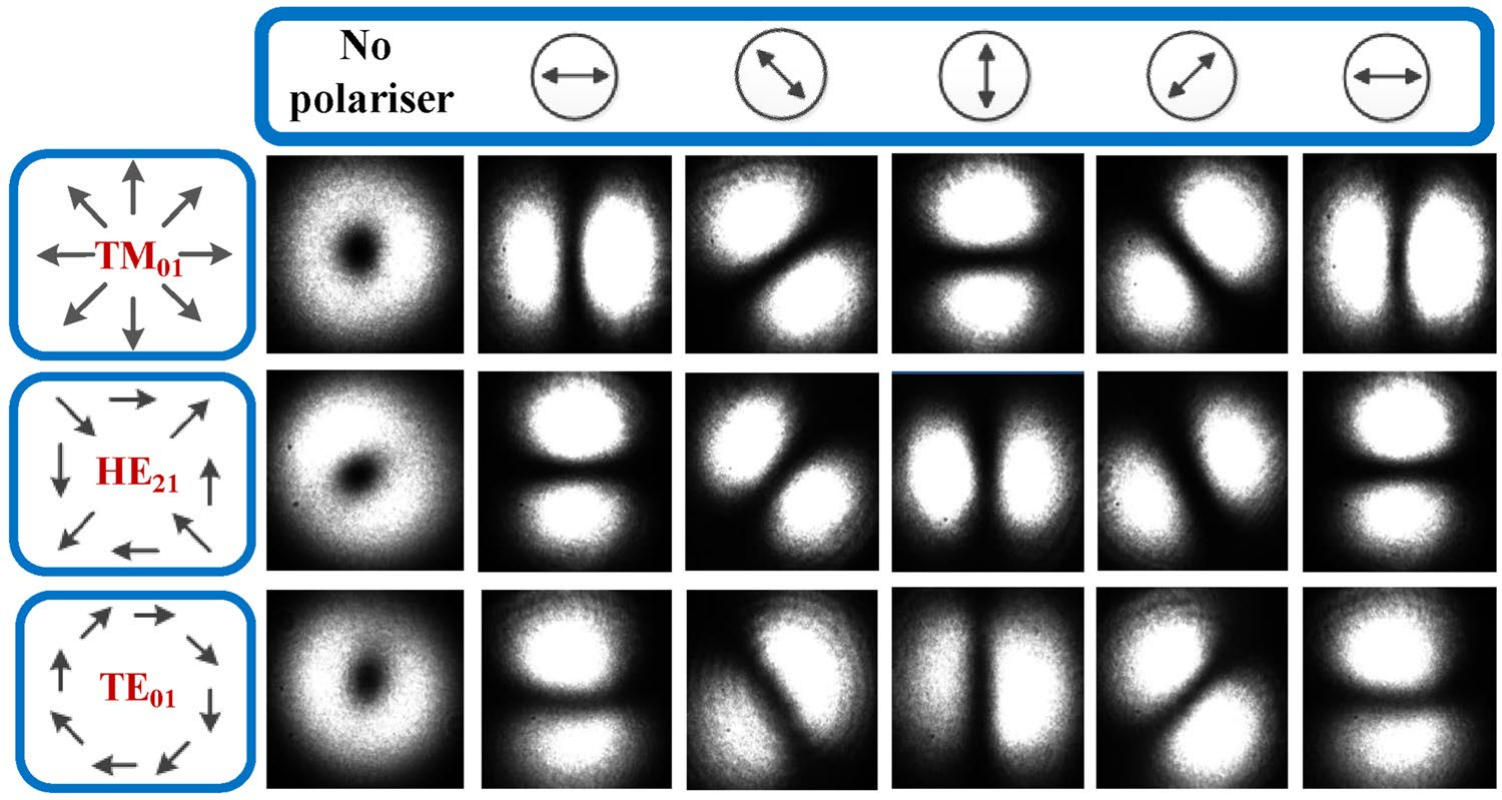
Identification of the TM_01_, HE_21_ and TE_01_ vector modes via a linear polariser.

The experimental setup for measuring the BGS of different vector modes is illustrated in Fig. 1. The upper branch of the setup (Fig. 1 area inside dotted box) shows the arrangement utilized for the excitation and detection of different vector modes in the FMF. In the arrangement, the output of the SMF is spliced into the input of FMF after which the fundamental mode (HE_11_ or LP_01_) is converted into the desired high-order vector mode by the LPFG. The latter scheme is reciprocal and thus also works in the opposite direction: any reflected power from vector modes in the FMF is converted back to the fundamental mode by the LPFG. Polarization controllers and mode strippers are inserted to maximize the conversion efficiency of the LPFG. The average power conversion efficiency from the fundamental mode to each of the three different vector modes using LPFG is measured to be 98.4%. The end facet of the FMF is projected onto a CCD camera in order to dynamically monitor the excited modes. The images of different vector modes (in the LP_11_ mode group) exhibit a similar doughnut shaped intensity profile as shown in the inset of Fig. 1. To discriminate and identify each vector mode, a polariser (i.e. analyzer) is placed in front of the CCD camera and rotated at different angles as illustrated in Fig. 2.

**Figure 3:**
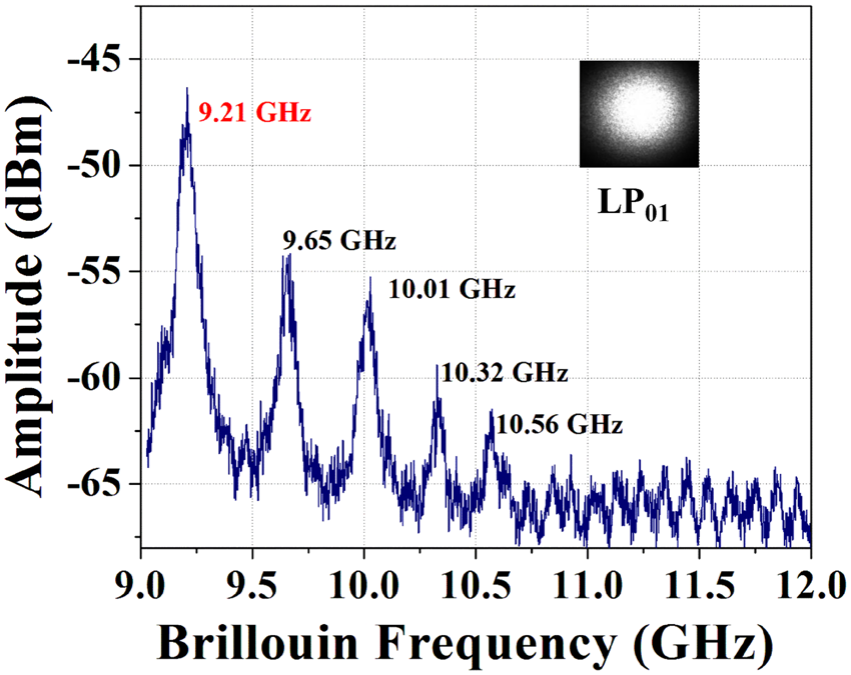
Full Brillouin gain spectra of the fundamental LP_01_ mode of FMF at 1550 nm.

In the experimental configuration (Fig. 1), a tunable laser with a 100-kHz linewidth is used as the light source. The 1550-nm CW light is split in two directions using a 50:50 single-mode fiber coupler. The upper path of the pump beam is first amplified to 25 dBm by an erbium doped fiber amplifier (EDFA) before being fed to the 50 m long FMF via an optical circulator (OC). The LPFG acts as the mode-converting device between the incident fundamental mode and the desired higher-order vector modes in the FMF (and vice-versa), as explained earlier. The reflected beam from the FMF contains a component at the same optical frequency as that of the incident beam (*ν*_*O*_), and includes a slightly red-shifted spectral component owing to nonlinear Brillouin scattering. The Brillouin reflected signal (i.e. Stokes signal) – of frequency *ν*_*O*_-*ν*_*B*_ where (*ν*_*B*_) is the Brillouin frequency shift – is then optically mixed with the lower path beam (acting as a local oscillator) and the corresponding optical beat signal is measured by a fast photodiode and electrical spectrum analyzer (ESA). Figure 3 shows the Brillouin spectrum obtained by launching a fundamental mode into the FMF and analyzing the backscattered Stokes signal trace using the ESA. The multiple peaks located at 9.21, 9.65, 10.01, 10.32 and 10.56 GHz correspond to the Brillouin scattering driven by various higher-order acoustic modes comprising both longitudinal and shear acoustic wave components^5^. In this study, our analysis focuses on the fundamental acoustic mode (centered around 9.21 GHz) as it is the dominant peak with the strongest Brillouin scattering response. Nonetheless, the methodology presented hereafter could be extended to other acoustic modes of interest.

## Results and Discussion

**Figure 4:**
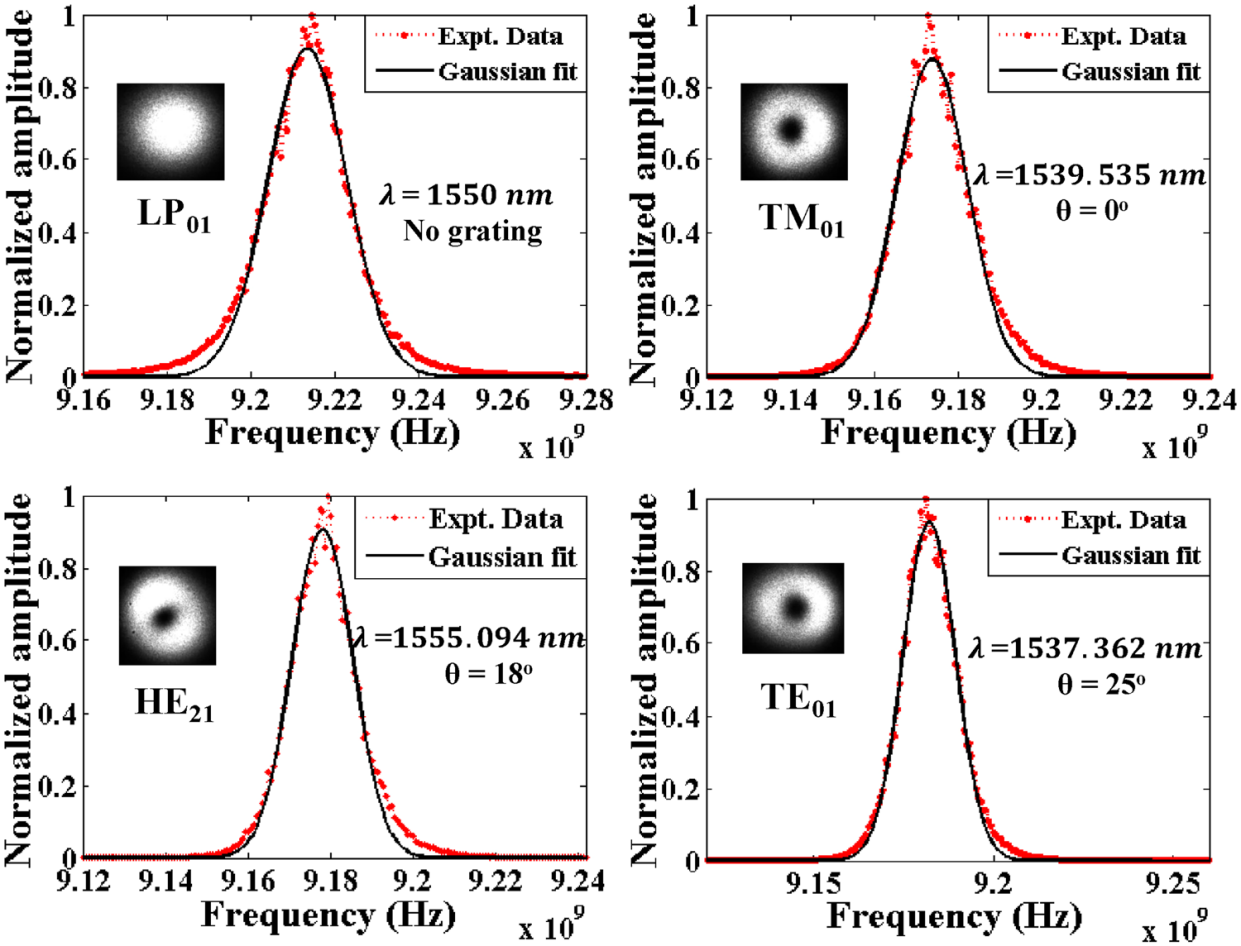
Measured Brillouin gain spectra for fundamental and high-order vector modes, and corresponding Gaussian fit curves.

The measured Brillouin gain spectra for the fundamental LP_01_ optical mode and the vector components of the LP_11_ mode group are presented in Fig. 4. To increase the signal to noise ratio of the BGS signal of each vector mode, ten sweeps were averaged before applying a Gaussian curve fit^21, 22^. Although the excitation of all these guided modes is possible at 1550 nm if the LPFG is set at the proper angle, the measured BGS shown in Fig. 4 were obtained at wavelengths slightly offset from the target 1550 nm wavelength (with the exception of the fundamental mode) due to imperfect fiber alignment on top of the LPFG’s grating lines when the latter is set at a non-zero angle. As a result, when pressure was applied on the fiber placed on top of the angled grating, it led to a non-uniformity of the grating period at some points along the ensuing LPFG. Therefore, in order to maximize the coupling efficiency of different vector modes, the phase-matching point was achieved by fine-tuning the resonance wavelength. Hence, in order to compare all BGS results at a common reference point, all the measurements (that were obtained at different wavelengths) were subsequently normalized to the same 1550 nm wavelength through a procedure detailed below.

### Wavelength normalization of Brillouin gain spectra at 1550 nm

The variation of Brillouin frequency shift (BFS) *ν*_*B*_ for acoustic modes in terms of acoustic velocity *V*_*a*_ and optical modes with ERIs (*n*_*eff*_) can be expressed as

1 $${\nu }_{B}=\frac{2{n}_{eff}}{\lambda }{V}_{a}$$

where *λ* is the pump wavelength. The value of acoustic velocity (*V*_*a*_) for germania-doped silica glass can be obtained through linear interpolation of the data based on the Makishima–Mackenzie model^23^. In this FMF, the maximum refractive index difference of ${\rm{\Delta }}n\approx 0.0442$ at the core-cladding interface thus corresponds to a velocity of 4876 m/s for the fundamental longitudinal acoustic mode^5^.

**Figure 5:**
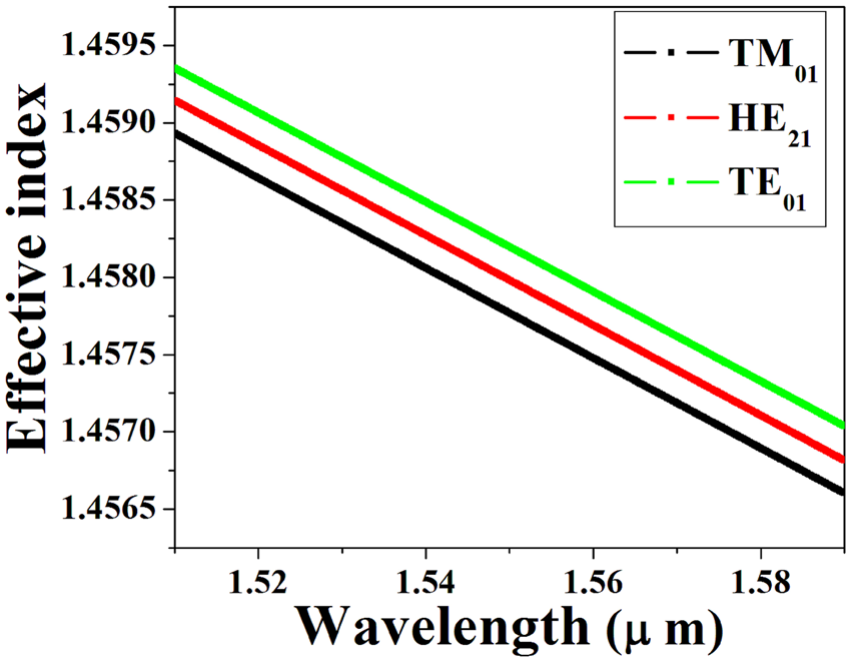
ERIs of the vector modes in the LP_11_ group calculated via FEM simulations based on the measured RIP of the FMF.

As seen from Eq. (1), using the latter value for *V*_*a*_ and the measured *ν*_*B*_, we can deduce the *n*_*eff*_ values of the guided modes based on their specific BFS values at a given wavelength *λ*. Now, in order to retrieve the ${n}_{eff}^{^{\prime} }$ values of the TE_01_, HE_21_ and TM_01_ modes at a common 1550 nm wavelength, the chromatic dispersion of their respective *n*_*eff*_ values [see Fig. 5] was taken into consideration via:

2 $${n}_{eff}^{^{\prime} }={n}_{eff}+(\frac{d{n}_{eff}}{d\lambda }){\rm{\Delta }}\lambda $$

Using Eq. (2) we are thus in a position to normalize the measurement of the BFS of different vector modes performed at different resonance wavelengths (*λ*) to the new wavelength (*λ*′ = 1550 nm) shifted by Δ*λ* = (*λ*′ − *λ*).

The Brillouin frequency shift *ν*_*B*_ of the BGS measured for the fundamental and high-order vector modes in the 50 m FMF are summarized in Table 1. It is notable that the BFS of the vector components of the LP_11_ mode group are separated by small but non-negligible shifts that enable the independent characterization of these vector modes. Subsequently, the effective refractive indices of the vector modes, as well as their corresponding separations (Δ*n*_*eff*_), were extracted from the *ν*_*B*_ measurements using Eq. (1). We note that the modal separations calculated via the BGS are almost two times larger than prior measurements^15, 19^. We attribute these discrepancies to two main factors: a) the longitudinal variations in the fiber’s refractive index profile (whose local effect on the fiber’s *n*_*eff*_ values are accentuated by the very large core-cladding index contrast), and to its non-negligible birefringence which is capable of inducing significant deviations (>10^−4^) in the ERI value of a vector mode^24^, b) the presence of a double-peak structure at the crest of the measured BGS (as shown in Fig. 4) – ostensibly related to polarization mode dispersion^24^ – introduces an unavoidable level of uncertainty in the curve fitting process. In fact, the double-peak is characterized by a spectral half-width of ~2 MHz which translates (via Eq. (1)) into a maximum error of ±3.2 × 10^−4^ in the corresponding effective index value. The latter magnitude of the error allows to bring into agreement our results in Table 1 based on the BFS, with earlier reported results performed on the same fiber but via other methods^15, 19^.

**Table 1:** Characteristics of the Brillouin gain spectra of vector modes.

Mode	Brillouin frequency shift *ν*_*B*_ (GHz)	Effective refractive index *n*_*eff*_	Modal separation Δ*n*_*eff*_	Brillouin gain linewidth Δ*ν*_*B*_ (MHz)	Brillouin gain threshold (dBm)
LP_01_	9.2136 ± 0.0003	1.46443 ± 4 × 10^−5^		23.54 ± 0.32	14.4
TE_01_	9.1820 ± 0.0003	1.45942 ± 5 × 10^−5^	(5.0 ± 0.1) × 10^−3^	18.02 ± 0.69	21.0
HE_21_	9.1783 ± 0.0003	1.45882 ± 5 × 10^−5^	(5.9 ± 0.9) × 10^−4^	18.83 ± 0.40	21.3
TM_01_	9.1742 ± 0.0003	1.45816 ± 5 × 10^−5^	(6.5 ± 0.7) × 10^−4^	21.01 ± 0.36	18.3

**Figure 6:**
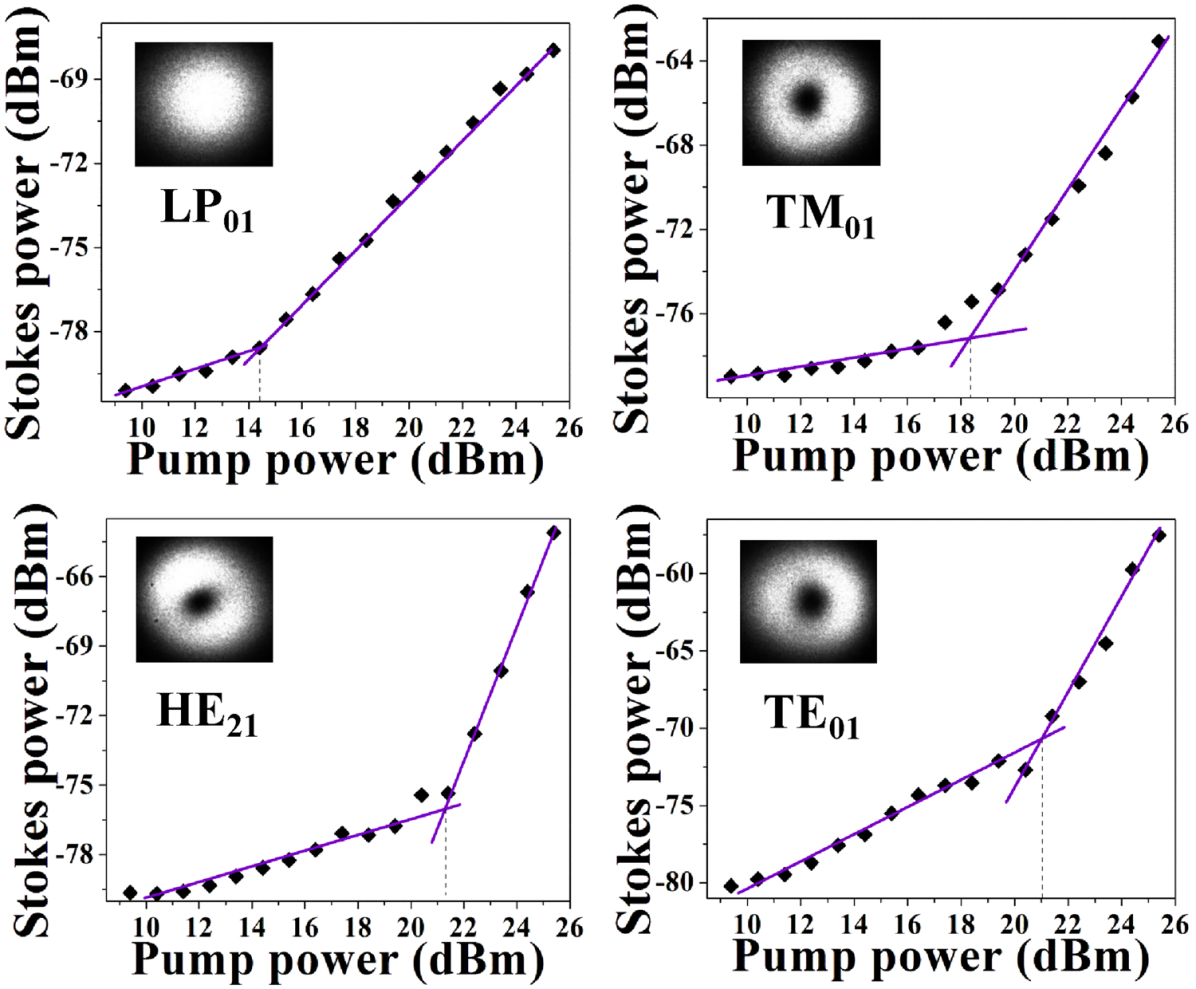
Brillouin threshold power of the LP_01_, TM_01_, HE_21_ and TE_01_ modes in the FMF.

Finally, we measured the Brillouin threshold for each vector mode by varying the pump power from 9 dBm to 25 dBm. Plots of the Stokes signal power as a function of pump input power are presented in Fig. 6. The Brillouin threshold of the vector modes are higher than that of the fundamental LP_01_ mode, and this difference is +4 dB for TM_01_ and +7 dB for HE_21_ and TE_01_. We suspect that the lower Brillouin threshold observed for the TM_01_ mode (compared to the TE_01_ and HE_21_ modes) can be explained by a polarization-mode-dependent photon-phonon coupling efficiency arising from the interplay of the different inhomogeneously polarized vector modes with the specific refractive-index profile of the fiber supporting the photo-acoustic interactions^25, 26^.

## Conclusion

Measurement of the Brillouin gain spectra of the vector modes in a FMF has been demonstrated for the first time using a simple heterodyne detection technique. Based on the measured Brillouin frequency shifts, we were able to extract the effective refractive indices of the vector modes in the FMF in a completely non-destructive manner. We predict that this new characterization method of individual vector modes will have an impact in both lightwave and fiber-optic sensing applications, which currently mostly rely on the scalar LP modes. Moreover, the ability of some specialty FMFs to lift the degeneracy of higher-order vector modes can be exploited in optical sensing (instead of their coupled LP modes counterpart), thereby bringing a larger number of independent states towards multi-parameter sensing. Because OAM mode-division multiplexing depends on the precise control of vector modes, it is also expected that the proposed method will help in the remote and non-destructive modal diagnostic of OAM fibers and their design optimization towards stable OAM modes transmission or in narrow-linewidth Brillouin FMF lasers.

## Methods

### Long period fiber grating as a mode converter

**Figure 7:**
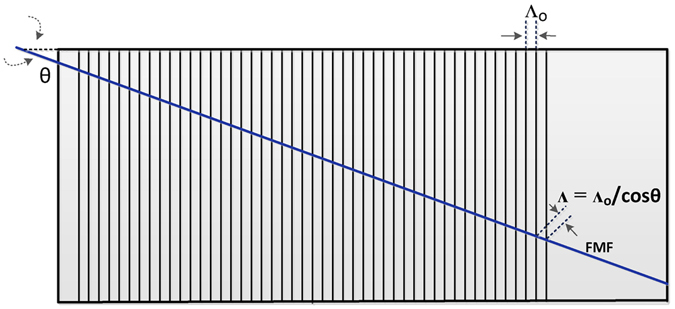
Positioning of the fiber at different angles w.r.t. LPFG (top view with angle *θ*) to vary the period.

The LPFG was devised using 200 *μm* diameter steel wires placed on top of a rectangular aluminum block (6 cm long and 4 cm wide). The wire diameter determines both the height and nominal pitch ${{\rm{\Lambda }}}_{\circ }$ of the grating^20^. As seen in Fig. 7, the bare fiber was placed at varying angles *θ* with respect to the grating in order to excite individual vector modes. The angles calculated to excite TM_01_, HE_21_ and TE_01_ were 0, 18 and 25 degrees respectively. The vector modes generated using LPFG were found to be stable over time and their modal purities (in dB) were calculated using the procedure outlined in ref. 27: 22.5 dB, 19.7 dB and 18.8 dB for the TM_01_, HE_21_ and TE_01_ modes respectively. The conversion efficiency of each vector mode was calculated by measuring the power transmitted at the output end of the fiber before and after the LPFG was used. Firstly, the power *P*_1_ was measured at the output end of the fiber without the LPFG. Then power *P*_2_ was measured after the vector mode generation when the LPFG was applied. The corresponding mode conversion efficiency in dB was calculated via^28^

3 $${\eta }_{dB}=10\,{\mathrm{log}}_{10}\,(\frac{{P}_{1}-{P}_{2}}{{P}_{1}})$$

Subsequently, the conversion efficiency in terms of percentage is given by

4 $${\eta }_{ \% }=(1-{10}^{\tfrac{{\eta }_{dB}}{10}})\times 100 \% $$

Proper identification of vector modes was ensured by using the polariser in front of the CCD camera (see Fig. 2) before recording measurements.
